# Safety of intermittent Pringle maneuver during minimally invasive liver resection in patients with hepatocellular carcinoma with and without cirrhosis

**DOI:** 10.1007/s00423-021-02361-z

**Published:** 2021-11-17

**Authors:** Santiago A. Ortiz Galindo, Philipp K. Haber, Christian Benzing, Felix Krenzien, Anna Riddermann, Oliver Frisch, Wenzel Schöning, Moritz Schmelzle, Johann Pratschke, Linda Feldbrügge

**Affiliations:** 1 Department of Surgery, Campus Charité Mitte and Campus Virchow-Klinikum, Charité Universitätsmedizin Berlin, Augustenburger Platz 1, 13353 Berlin, Germany; 2 Berlin Institute of Health, Charité - Universitätsmedizin Berlin, Charitéplatz 1, 10117 Berlin, Germany

**Keywords:** Hepatocellular carcinoma, Pringle maneuver, Cirrhosis, Liver resection, Minimally invasive surgery

## Abstract

**Purpose:**

The aim of this study was to analyze the impact of minimally invasive intermittent Pringle maneuver (IPM) on postoperative outcomes in patients with hepatocellular carcinoma (HCC) and liver cirrhosis.

**Methods:**

In this retrospective cohort study, we evaluated the safety of IPM in patients with HCC who underwent minimally invasive liver resection during five years at our center. Factors influencing the use of IPM were examined in univariate and multivariate regression analysis. Cases with use of IPM (IPM) and those without use of IPM (no IPM) were then compared regarding intraoperative and postoperative outcomes after propensity score matching (PSM) for surgical difficulty.

**Results:**

One hundred fifty-one patients underwent liver resection for HCC at our center and met inclusion criteria. Of these, 73 patients (48%) received IPM with a median duration of 18 min (5–78). One hundred patients (66%) had confirmed liver cirrhosis. In multivariate analysis, patients with large tumors (≥ 3 cm) and difficult tumor locations (segments VII or VIII) were more likely to undergo IPM (OR 1.176, *p* = 0.043, and OR 3.243, *p* = 0.001, respectively). After PSM, there were no differences in intraoperative blood transfusion or postoperative complication rates between the IPM and no IPM groups. Neither did we observe any differences in the subgroup analysis for cirrhotic patients. Postoperative serum liver function tests were not affected by the use of IPM.

**Conclusions:**

Based on our findings, we conclude that the use of IPM in minimally invasive liver resection is safe and feasible for patients with HCC, including those with compensated liver cirrhosis.

**Supplementary Information:**

The online version contains supplementary material available at 10.1007/s00423-021-02361-z.

## Introduction

Hepatic inflow occlusion during parenchymal resection, commonly called the Pringle maneuver [1], has been shown to reduce blood loss and improve short-term surgical outcome [2–4], but has also been suspected to cause liver injury due to ischemia–reperfusion damage, based on findings in animal experiments [5, 6]. Several retrospective studies and a few small randomized controlled trials have been published about the efficacy and safety of continuous or intermittent Pringle maneuver (IPM). Nevertheless, the use of Pringle maneuver remains controversial even to this day, with contradictory findings concerning the effects on blood loss and resulting liver injury [7, 8].

Hepatocellular carcinoma (HCC) is the most common primary liver cancer and presents a challenge to liver surgeons, as it most often occurs in cirrhotic livers. Minimally invasive techniques have been widely adopted for minor and increasingly for major liver resection, including for HCC. Several recent studies, including one randomized controlled trial, have compared laparoscopic with open liver resection selectively for HCC patients and have coherently found shorter hospital stay, lower or similar complication rates, and comparable oncologic outcomes [9–11].

Data on safety and efficacy of the Pringle maneuver in minimally invasive surgery are scarce. Most available studies are technical, focusing on partial inflow occlusion or comparing intermittent to continuous occlusion [12–14]. Regarding the use of the Pringle maneuver in cirrhotic livers for HCC resections, findings have been controversial. On the one hand, risk for bleeding is higher in cirrhotic patients, and some studies have found reduction of blood loss [14, 15]. On the other hand, cirrhotic livers may be more vulnerable to ischemia–reperfusion injury [16]. In patients with HCC, who are commonly also diagnosed with liver cirrhosis, the Pringle maneuver has been mostly demonstrated to be safe without a higher incidence of postoperative complications such as postoperative liver failure [4, 17].

The aim of this study was therefore to determine the safety of IPM in minimally invasive liver resection for HCC, with a focus on patients with underlying liver cirrhosis. This topic is highly relevant in times of declining transplantation rates due to organ scarcity, along with a continuous expansion of indication to surgery, including more patients with advanced cirrhosis. Few studies have analyzed outcomes of IPM in minimally invasive liver surgery, and to our knowledge, no publication focused on IPM in minimally invasive liver resections in the special group of cirrhotic HCC patients.

## Methods

### Study design

In this retrospective cohort study, we analyzed data of all consecutive patients who underwent minimally invasive liver resection for HCC at the Department of Surgery, Campus Charité Mitte and Campus Virchow-Klinikum, Charité—Universitätsmedizin Berlin between January 2015 and December 2020.

The aim was to evaluate the safety of IPM in minimally invasive surgery for HCC, with a focus on patients with underlying liver cirrhosis. Resections that included the use of IPM (IPM) were compared to those performed without IPM (no IPM) with regard to perioperative complications and liver function. A subgroup analysis was performed for patients with liver cirrhosis. To control for potential selection bias, we first determined factors leading to the use of IPM in univariate and multivariate analysis and then performed a propensity score-based matching (PSM) based on these findings. All included patients gave informed consent to the collection of their personal and medical data and its use for research purposes. All data were collected, stored, and processed according to the General Data Protection Regulation and local data protection laws. Cases that lacked conclusive information about use or duration of IPM were excluded. The study was conducted in accord with the ethical standards of the Helsinki Declaration of 1975. The Charité institutional review board approved of the study (EA2/006/16 and EA4/084/17).

### Surgical techniques of minimally invasive liver resection and hepatic inflow occlusion

Different minimally invasive access strategies were used in our cohort: Multi-incisional laparoscopic surgery (MILS) was the most common laparoscopic approach, followed by multi-incisional robotic surgery, and, in the early years, hand assisted laparoscopic surgery (HALS) or single-incision laparoscopic surgery (SILS). Surgical techniques of laparoscopic and robotic liver resection are described in details elsewhere [18–20]. In both laparoscopic and robotic surgery, ultrasound was routinely performed intraoperatively to confirm the exact tumor location, borders, and proximity to vascular and biliary structures as well as to rule out further intrahepatic lesions.

IPM was performed using a soft, 3-mm-wide polyethylene terephthalate ribbon that was positioned around the hepatoduodenal ligament before both ends were threaded through a 5-mm trocar on the patient’s left side and externally, through a plastic tube (tourniquet) of approximately 10-cm length. The ends could then be tightened and secured in their position with a clamp at any moment for full inflow occlusion. The anesthesiologist kept a precise log with the respective starting and release times of IPM. Duration of IPM never exceeded 15 min, with a minimum of 5 min reperfusion time between two maneuvers. The decision to use or refrain from IPM was made by the surgeon according to his perceived risk of bleeding. For parenchymal dissection in laparoscopic procedures, we used a water-jet dissector (ERBEJET® 2, Erbe, Tübingen) or an ultrasonic dissector (cavitron ultrasonic surgical aspirator, CUSA, Integra LifeSciences, Saint Priest, France). Other devices, used mostly in combination, were ultrasonic surgical devices with clamp tips such as Harmonic Ace® (Johnson & Johnson, Norderstedt) or THUNDERBEAT (Olympus, Hamburg) or endoscopic linear cutter staplers (Echelon, Johnson & Johnson, USA). In robotic resections, a modified clamp crush technique was applied, using Harmonic ACE®, while large vessels were either clipped or transected using staplers, as in conventional laparoscopic surgery.

### Perioperative clinical management and clinical outcome parameters

We determined common patient characteristics such as age, sex, body mass index (BMI), and the general physical status using the American Society of Anesthesiologists’ Physical Status Classification (ASA score). Underlying liver disease was characterized by etiology; the preoperative model of end-stage liver disease (MELD) score and the histological stage of liver fibrosis with cirrhosis defined as a stage 4 according to Desmet et al. [21] and classified in clinical severity according to the Child Pugh Score. HCC were classified according to size, number of nodules, Milan Criteria [22] and the Barcelona clinic liver cancer (BCLC) classification [23]. Furthermore, the extent (major vs. minor) and the complexity of liver resection as assessed by IWATE classification [24] were determined. Major liver resection was defined as a resection of three or more continuous segments.

After PSM, cases with and without use of IPM were compared with respect to the duration of surgery, need for blood transfusion, length of stay in the hospital after surgery (LOS), length of stay in the intensive care unit (ICU-LOS), postoperative complications (according to Clavien-Dindo classification [25]), textbook outcome [26], post-hepatectomy liver failure (PHLF) as graded according to the International Study Group of Liver Surgery (ISGLS) criteria [27], and resection status (R-classification by pathology). Liver function and hemostatic parameters were assessed by perioperative serum levels of aspartate aminotransferase (AST), aspartate alaninotransferase (ALT), bilirubin, platelet counts, partial thromboplastin time (PTT), and the international normalized ratio (INR). For this, we analyzed one preoperative time point, the first postoperative day (POD1), and the day of or before hospital discharge (pre-discharge). Intraoperative arterial blood gas analysis was measured at the beginning, in the middle and at the end of surgery.

### Statistics

Categorical data were analyzed using a Pearson’s chi-square test and are presented as frequencies and percentages. Continuous data were analyzed by Mann–Whitney *U* test and presented with median and range (minimum–maximum). Logistic regression analysis was used to find independent predicting factors for the use of IPM, using variables that were significantly associated with use of IPM in univariate analysis for a subsequent multivariate analysis. A propensity score matching was performed using the “MatchIt” package from R 4.0 without replacement, using nearest neighbor method, with a caliper of 0.1, with dependent variables: tumor location in segment VII or VIII, tumor size ≥ 3 cm, ASA score ≥ 3, and liver cirrhosis. A second propensity score matching was performed for the subgroup analysis only in patients with liver cirrhosis as previously explained, without liver cirrhosis as a dependent variable. A *p* value < 0.05 was considered statistically significant. IBM SPSS Statistics Version 26 and R version 4.0 were used for all statistics. R version 4.0 and GraphPad Prism 5 were used for graphs.

## Results

### Baseline characteristics and surgical techniques

Between February 2015 and December 2020, 151 minimally invasive liver resections for HCC were performed at our center and met inclusion criteria. With regard to the etiology of the underlying liver disease, we observed 64 cases (42%) of viral hepatitis and 29 (19%) of alcoholic liver disease. Only in 12 cases (8%), the HCC had reportedly occurred in healthy livers. Liver cirrhosis, as determined by histopathology, was present in 100 cases (66%). In 40 cases (27%), HCC was multifocal. Tumor sizes varied with a median of 3 cm (0.3–18 cm) diameter of the largest nodule. The majority of surgeries (78%) were minor resections. Median surgical difficulty as assessed by the IWATE classification was seven, which is considered “advanced” [24]. Thirty-two cases (21%) were operated using the da Vinci Xi surgical system, while the remaining liver resections were conventional laparoscopies. In almost half of the surgeries (48%), IPM was applied during parenchymal resection. The median duration of inflow occlusion among these cases was 18 min (5–78). Baseline characteristics of the patients and tumors and details of the surgical techniques are summarized in Table 1, both for the entire cohort and for matched groups with and without IPM.

**Table 1:** Minimally invasive liver resection for hepatocellular carcinoma (HCC). Baseline characteristics, classifications of liver disease and tumor stage, and surgical techniques for whole cohort and propensity score matched groups with and without use of intermittent Pringle maneuver (IPM)

	Whole cohort	Post -PSM		
	*n* = 151	IPM *n* = 46	No IPM *n* = 46	*p*
Age (years)	68 (19–86)	69 (49–85)	68 (19–86)	0.484
Sex (female)	38 (25%)	10 (22%)	11 (24%)	1.000
ASA ≥ 3	90 (60%)	30 (65%)	30 (65%)	1.000
Body mass index (kg/m^2^)	26.8 (18–44)	26.8 (20–40)	27.45 (18–37)	0.885
Underlying liver disease^a^				0.234
None	12 (8%)	4 (9%)	1 (2%)	
Non-alcoholic liver disease (NASH)	14 (9%)	5 (11%)	4 (9%)	
Alcoholic liver disease	29 (19%)	10 (23%)	12 (27%)	
Other/cryptogenic	25 (16%)	9 (20%)	4 (9%)	
Viral hepatitis	64 (42%)	16 (37%)	24 (53%)	
Hepatitis B	23 (36%)	4 (25%)	9 (38%)	0.408
Hepatitis C	40 (63%)	12 (75%)	15 (62%)	
Co-infection hepatitis B + C	1 (1%)			
Liver fibrosis stage^b^				0.973
0	4 (3%)	1 (2%)	1 (2%)	
I	9 (6%)	3 (7%)	3 (7%)	
II	17 (11%)	4 (9%)	3 (7%)	
III	18 (12%)	3 (7%)	5 (11%)	
IV (liver cirrhosis)	100 (66%)	34 (76%)	34 (74%)	
Child Pugh score				1.000
A	94 (94%)	32 (94%)	31 (91%)	
B	6 (6%)	2 (6%)	3 (9%)	
Preoperative MELD score	8 (5–24)	8 (6–24)	8 (6–17)	0.383
Tumor size (cm)^c^	3 (0.3–18)	3.1 (0.6–14)	3 (0.3–9.5)	0.207
Multifocal HCC	40 (27%)	8 (17%)	9 (20%)	1.000
Outside of Milan criteria	49 (33%)	15 (33%)	12 (26%)	0.647
BCLC 0-A	131 (87%)	44 (96%)	38 (83%)	0.090
Extent of resection				0.807
Major (≥ 3 segments)	33 (22%)	12 (26%)	10 (22%)	
Minor (< 3 segments)	118 (78%)	34 (74%)	36 (78%)	
IWATE score	7 (1–12)	8 (2–11)	7 (2–12)	0.369
Surgical technique				1.000
Robotic	32 (21%)	9 (20%)	10 (22%)	
Laparoscopic	119 (79%)	37 (80%)	36 (78%)	
Use of intermittent Pringle maneuver	73 (48%)	46 (100%)	0	
Total duration of Pringle maneuver (minutes)	18 (5–78)	19.5 (5–78)	0	-

Data presented as number (percent) for categorical or median (minimum–maximum) for continuous variables

^a^Seven cases with missing information about underlying liver disease

^b^According to Desmet classification of liver fibrosis. Three cases with missing information about fibrosis stage

^c^Largest lesion in case of multifocal tumor

*MELD* model of end stage liver disease, *BCLC* Barcelona clinic liver cancer staging

### Use of IPM in minimally invasive HCC resections

**Fig. 1:**
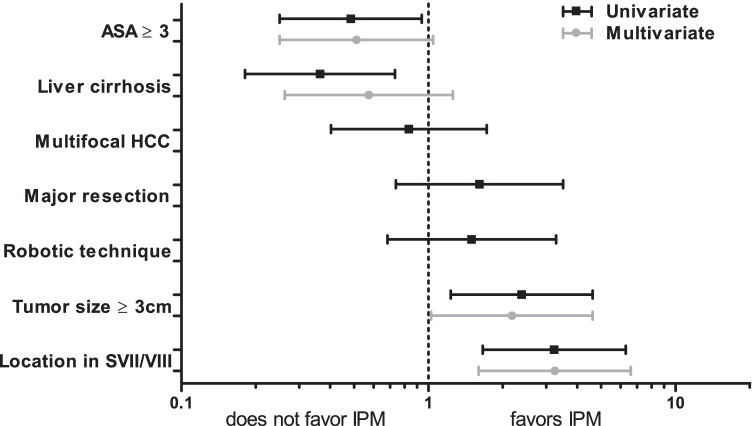
Factors influencing the use of intermittent Pringle maneuver (IPM) in minimally invasive liver resection for hepatocellular carcinoma (HCC)

Before analyzing the impact of IPM on perioperative outcome, we aimed to determine potential biases by finding factors that impact the surgeon’s decision to use IPM. Patient, tumor, and surgery-related characteristics were considered. In univariate analysis, we found that IPM was used less frequently in patients who have a poor general status according to ASA as well as in patients with liver cirrhosis (OR 0.485 (0.250–0.939), *p* = 0.032 and OR 0.364 (0.181–0.732), *p* = 0.005, respectively). On the other hand, IPM was more likely to be applied in patients with large tumors (OR 2.382 (1.230–4.612), *p* = 0.010) and those with tumors located in segments VII or VIII, scoring highest in IWATE difficulty score for tumor location (OR 3.228 (1.658–6.283), *p* = 0.001). Multifocal disease, extent of resection (major vs. minor resection), or the type of surgical access approach (robotic vs. laparoscopic) did not seem to influence the use of IPM (Fig. 1, black bars).

After multivariate analysis using the variables that were significantly associated with the use of IPM in univariate analysis, only tumor size (OR 1.176 (1.026–4.613), *p* = 0.043) and difficult tumor location (OR 3.243 (1.596–6.589), *p* = 0.001) independently predicted the use of IPM during HCC resection (Fig. 1, gray bars). Neither a high ASA score nor underlying liver cirrhosis was significantly associated with avoiding the use of IPM. These findings suggest that technical difficulty is crucial for deciding about the use of IPM and that liver cirrhosis itself does not appear to discourage from applying IPM.

### Postoperative complications after minimally invasive HCC resection with and without IPM

To compare perioperative outcomes of minimally invasive HCC resection with and without IPM, we performed a PSM to control for potential selection biases, including the variables that were different in our univariate and/or multivariate analysis. After matching, each group comprised 46 patients. Covariates are balanced well after PSM, as displayed in Supplementary Fig. 1.

Before matching, the median duration of surgery was significantly longer when IPM was applied (difference of 48 min or 25% longer, *p* = 0.001). After PSM, there was no significant difference in duration of surgery, indicating that IPM itself does not prolong surgery, but rather that cases including IPM use were more difficult. The rate of intraoperative red blood cell transfusion in our cohort was 6% with no significant differences between groups before or after PSM. Intraoperative use of IPM did not impact complication rates; overall postoperative morbidity in the whole cohort was 39% with a 15% incidence of severe complication (Clavien-Dindo ≥ 3a), with similar rates of textbook outcome, PHLF, and bile leakage. Patients spent one night (median) on the ICU, and six days in the hospital after surgery, irrespective of undergoing IPM during resection. Ninety-five percent of resections were graded as R0 by the pathologist with no difference between groups. All short-term outcome criteria with and without use of IPM, before and after matching, are detailed in Table 2.

**Table 2:** Intraoperative and postoperative outcome criteria after minimally invasive liver surgery for hepatocellular carcinoma (HCC) with vs. without use of intermittent Pringle maneuver (IPM), before and after propensity score matching (PSM)

	*Pre-PSM*			*Post-PSM*		
	IPM *n* = 73	No IPM *n*** = **78	*p*	IPM *n* = 46	No IPM *n* = 46	*p*
Duration of surgery (min)	240 (102–491)	192 (49–461)	*0.001*	231 (102–491)	196.5 (49–461)	0.069
Red blood cell transfusion	6 (8%)	3 (4%)	0.315	5 (11%)	3 (7%)	0.714
Textbook Outcome ^a^	56 (77%)	65 (83%)	0.308	32 (70%)	37 (80%)	0.229
Postoperative complications ^b^	26 (36%)	33 (42%)	0.400	21 (46%)	24 (52%)	0.532
Severe complications ^c, d^	12 (16%)	10 (13%)	0.529	9 (20%)	8 (17%)	0.788
PHLF	1 (1%)	3 (4%)	0.621	1 (2%)	2 (4%)	1.000
Grade A		2 (75%)			2 (100%)	
Grade B	1 (100%)	1 (25%)		1(100%)		
Bile leak	7 (10%)	6 (8%)	0.775	4 (9%)	4 (9%)	1.000
Post-hepatectomy hemorrhage	1(1%)	2 (3%)	1.000	1 (2%)	1 (2%)	1.000
Mortality^a^	1 (1%)		0.300	1 (2%)		1.000
LOS, ICU (days)	1 (0–43)	1 (0–6)	0.298	1 (0–43)	1 (0–5)	0.223
LOS, hospital (days)	6 (3–61)	6 (3–26)	0.345	6 (3–61)	7 (3–26)	0.686
R0	68 (93%)	75 (96%)	0.484	42 (91%)	44 (96%)	0.677
Conversion rate		1 (1%)	1.000		1 (2%)	1.000

Data presented as number (percent) for categorical or median (minimum–maximum) for continuous variables. *PHLF* post-hepatectomy liver failure [27], *LOS* length of stay, *ICU* intensive care unit, *R0* resection status (no residual tumor)

^a^Defined as no severe complication (≥ 3 according to Clavien-Dindo classification of postoperative complications) [25]; no intraoperative complication ≥ 2 (according to the Oslo classification of intraoperative complications [28]); R0 resection status achieved; no re-admission in 30 days post-discharge; no in-hospital mortality; absence of bile leak grades B or C[26]

^b^Within 90 days after surgery

^c^ ≥ 3a according to Clavien-Dindo classification of postoperative complications[25]

To examine the safety of using IPM in liver cirrhosis, we compared the same short-term outcome parameters between IPM and no IPM in the subgroup of cirrhotic patients, selectively, after performing PSM (Table 3). Before matching, similar to the entire, unmatched cohort, there was a (non-significant) trend towards longer duration of surgery in cases with use of IPM, while all other outcome parameters were similar between the groups. After PSM, there were no significant differences between IPM and no IPM (34 patients per group).

**Table 3:** Intraoperative and postoperative outcome criteria after minimally invasive liver surgery for hepatocellular carcinoma (HCC) in the subgroup of patients with liver cirrhosis, with vs. without use of intermittent Pringle maneuver (IPM), before and after propensity score matching (PSM)

	*Pre-PSM*			*Post-PSM*		
	IPM *n* = 40	No IPM *n* = 60	*p*	IPM *n* = 34	No IPM *n* = 34	*p*
Duration of surgery (min)	222 (102–455)	187 (49–461)	0.054	218 (102–455)	189 (49–461)	0.194
Red blood cell transfusion	4 (10%)	2 (3%)	0.214	3 (9%)	2 (6%)	1.000
Textbook outcome^a^	32 (80%)	50 (83%)	0.671	26 (77%)	26 (77%)	1.000
Postoperative complications^b^	12 (30%)	23 (38%)	0.392	11 (32%)	14 (41%)	0.615
Severe complications^c,d^	4 (10%)	7 (12%)	0.794	4 (12%)	6 (18%)	0.493
PHLF	1 (3%)	2 (3%)	1.000	1 (3%)	1 (3%)	1.000
Grade A		1 (50%)			1 (100%)	
Grade B	1 (100%)	1 (50%)		1 (100%)		
Bile leak	2 (5%)	3 (5%)	1.000	2 (6%)	1 (3%)	1.000
Post-hepatectomy hemorrhage	1 (3%)	1 (2%)	1.000	1 (3%)		1.000
Mortality^a^	1 (3%)		0.400	1 (3%)		1.000
LOS, ICU (days)	1 (0–43)	1 (0–6)	0.951	1 (0–43)	1 (0–4)	0.815
LOS, hospital (days)	6 (3–42)	6 (3–26)	0.507	6 (3–42)	7 (3–26)	0.218
R0	37 (93%)	57 (95%)	0.681	31 (91%)	31 (91%)	1.000
Conversion rate		1 (2%)	1.000		1 (3%)	1.000

Data presented as number (percent) for categorical or median (minimum–maximum) for continuous variables. *PHLF* post-hepatectomy liver failure [27], *LOS* length of stay, *ICU* intensive care unit, *R0* resection status (no residual tumor)

^a^Defined as no severe complication (≥ 3 according to Clavien-Dindo classification of postoperative complications) [25]; no intraoperative complication ≥ 2 (according to the Oslo classification of intraoperative complications [28]); R0 resection status achieved; no re-admission in 30 days post-discharge; no in-hospital mortality; absence of bile leak grade B or C[26]

^b^Within 90 days after surgery

^c^ ≥ 3a according to Clavien-Dindo classification of postoperative complications[25]

Subgroup analyses were also performed for cases of major resection and long total duration of IPM (≥ 30 min) and showed no differences between IPM and no IPM, except for a longer duration of surgery in patients who underwent long IPM (Supplementary Tables 1 and 2).

### Perioperative liver function in patients with and without undergoing IPM

**Fig. 2:**
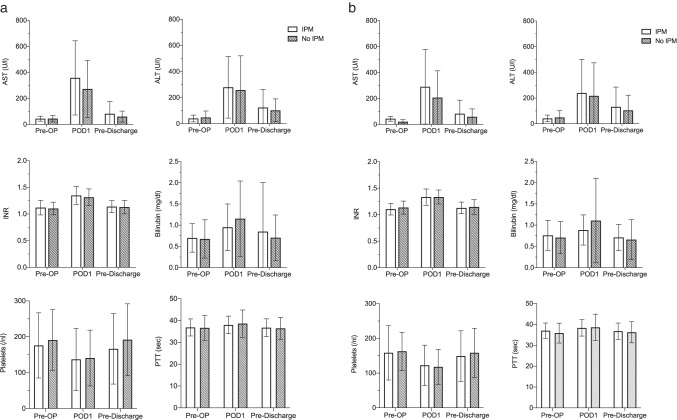
Laboratory liver function tests after minimally invasive liver surgery for hepatocellular carcinoma (HCC) with vs. without intermittent Pringle maneuver (IPM). **a** All patients (after propensity score matching). **b** Subgroup of patients with liver cirrhosis (after propensity score matching)

Serum concentrations of commonly used markers for liver function, AST, ALT, INR, and bilirubin, were all elevated on the first postoperative day (POD1) after liver resection (Fig. 2). While bilirubin decreased to preoperative levels by the time of discharge, AST, ALT, and INR remained slightly elevated, albeit below clinically relevant concentrations. AST shows a tendency to a higher increase postoperatively in patients who undergo IPM, when comparing IPM with no IPM (after PSM, including cirrhotic and non-cirrhotic patients, *p* = 0.072). However, at the time of discharge from hospital, no differences between both groups are longer measurable. ALT, bilirubin, INR, PTT, and platelet counts are similar in both groups at all time points (Fig. 2a).

When analyzing the subgroup of patients with liver cirrhosis, there was no significant difference in serum liver function tests in matched cohorts between IPM and no IPM (Fig. 2b). Postoperative AST levels did not correlate with the duration of IPM in those patients who received IPM, neither the entire group nor in the subgroup of cirrhotic patients (Supplementary Fig. 2).

Intraoperative arterial blood gas analysis showed a temporarily significantly enhanced acidosis in cases of IPM, a difference that was no longer visible at the end of surgery. Lactate levels at the end of surgery were slightly higher in patients who underwent IPM. The difference in pH, but not in lactate, was also visible in the subgroup of cirrhotic patients (Supplementary Fig. 3).

## Discussion

In this retrospective cohort study, we show that IPM during minimally invasive liver resection does not increase perioperative complication rates or risk for liver failure in patients with HCC, including those with liver cirrhosis.

As minimally invasive approaches have become the standard in liver surgery in experienced centers, it is essential to continuously analyze details of the surgical strategies and refine our techniques accordingly. While in the early years, minimally invasive approaches were reserved for benign lesions, minor resections, and mostly healthy patients, indications have been extended over time and now include major resections and patients with substantial liver and systemic disease, such as patients with HCC and underlying liver cirrhosis [9, 29]. We have observed the same development at our own center in the course of ten years and today perform all extents and indications of liver resections minimally invasively, with the exception of surgeries that include vascular or biliary reconstructions [20, 30].

From early on, we have liberally applied a laparoscopic version of the IPM that was well-known from open liver resection and have not noticed complications. However, with more difficult cases and increasingly diseased patients allocated to minimally invasive surgery, it is important to study the safety of IPM objectively, especially as the topic has been discussed so controversially over the years. There are important confounders to consider: We, as most others, use IPM selectively, in roughly 50% of the cases, and these cases are more likely to be difficult, introducing a relevant selection bias. We therefore tested which factors play a role in the surgeon’s decision. In multivariate analysis, the only independent predictors were large tumor size and difficult tumor location, while liver cirrhosis did not seem to discourage from IPM. We then performed a propensity score matching including any factors we had found that may impact the decision for or against IPM. Of course, although controlled for by PSM, a certain heterogeneity in our patient cohort, such as variable tumor size and tumor locations, must be taken into consideration for the interpretation of our data.

In the matched cohorts, transfusion rates were comparable with and without IPM, as were postoperative complication rates and all other outcome criteria, with the only exception of pronounced intraoperative acidosis and elevated lactate at the end of surgery. Interestingly, after PSM, there was no difference in liver function tests after surgery at any time point. In the unmatched cohort, transaminase elevation on the first postoperative day was more pronounced in cases of IPM. This indicates that temporary increase in liver cell damage is likely not enhanced by IPM itself, but other factors, such as extent and technical difficulty of resection. Importantly, in the group of patients with liver cirrhosis, there was also no difference in postoperative complications or liver function with regard to use of IPM. Subgroup analysis was also performed for patients with major resection and those cases where IPM duration exceeded 30 min, without finding differences in postoperative outcomes. Of note, we do not report the estimated blood loss as an outcome parameter, but instead focus on the need for transfusion as the best surrogate marker for blood loss, as it denotes those cases with clinically relevant bleeding. In our experience, estimated blood loss can be inaccurate, especially in cases of laparoscopy, where sometimes lavage fluid may remain intra-peritoneally and blood loss is generally low, leading to misleading calculations.

Especially in earlier years of open liver surgery, negative effects of IPM on liver function by ischemia reperfusion injury were postulated, stated as most relevant in patients with pre-existing liver cirrhosis [15]. Two randomized controlled trials that date back more than ten years showed no benefit of IPM and concluded that it should be avoided [31, 32]. However, more recently, an RCT from one of these centers, that included only HCC patients, could not confirm these findings and saw no increase in complications [17]. Several other recent studies report safe use of IPM, some with signs of improved outcomes in patients with HCC in cirrhosis [2, 33]. Our own results corroborate these more recent findings on the safety of IPM. The change in the risk assessment of IPM over the years may well be due to other advances in liver surgery, improving outcomes in general, especially the increased use of minimally invasive approaches.

With regard to minimally invasive IPM, there are several new studies, particularly discussing specific techniques, mostly hemi-hepatic inflow occlusion [12]. One recent study used a comparable technique to ours and similarly concluded that intermittent total hepatic inflow occlusion was safe and feasible in laparoscopic liver resection. However, they did not specify the extent of resection in their cohort, making a further comparison with our results difficult [13]. In our study, we focus on the short-term surgical outcomes of minimally invasive HCC resection. However, there is also still debate about long-term, oncological effects: The Pringle maneuver has been suggested to impair oncological outcomes after HCC resection [34, 35], while other studies have shown that IPM is safe without increased risk of early or long-term HCC recurrence [3, 17, 36]. When comparing laparoscopic to open liver resection for HCC in a previous study, we did not find any difference in long-term survival, but did not focus on the use of IPM [37]. Currently, with a short follow-up period and relatively small numbers, especially in the subgroup of patients with liver cirrhosis, it is beyond the scope of this analysis to assess the impact of IPM on oncological long-term outcome, but this question should be followed up and addressed in the future.

In our study, we find that IPM is safe in minimally invasive liver surgery for HCC in cirrhosis. However, as we do not see a relevant decrease in postoperative complication rates or transfusion rates, we cannot conclude that IPM is efficient and no general recommendation for the use of IPM in every case can follow from our findings. This was also not the aim of our study and would have to be examined in a different design, preferably including randomization.

## Conclusion

We propose that the use of IPM is a safe and useful tool in minimally invasive liver surgery, and does not compromise postoperative liver function, in patients with hepatocellular carcinoma including those with compensated liver cirrhosis.

## Supplementary Information

Below is the link to the electronic supplementary material.

Supplementary file1 (DOCX 11908 KB)

## Data Availability

The data presented are available on request from the corresponding author.
