# Supplementary material for: Safety of intermittent Pringle maneuver during minimally invasive liver resection in patients with hepatocellular carcinoma with and without cirrhosis
Source: Langenbecks Arch Surg. 2021 Nov 17;407(1):235–44. doi: 10.1007/s00423-021-02361-z (PMC8847278; doi:10.1007/s00423-021-02361-z)
Supplement: Supplementary file 1 — Supplementary file1 (DOCX 11908 KB) [file 423_2021_2361_MOESM1_ESM.docx]

**Article Name:** Safety of intermittent Pringle maneuver during minimally invasive liver resection in patients with hepatocellular carcinoma with and without cirrhosis

**Journal Name:** Langenbeck’s Archives of Surgery

**Authors:** Santiago A. Ortiz Galindo^1^, Philipp K. Haber^1^, Christian Benzing^1^, Felix Krenzien^1,2^, Anna Riddermann^1^, Oliver Frisch^1^, Wenzel Schöning^1^, Moritz Schmelzle^1^, Johann Pratschke^1^, Linda Feldbrügge^1,2^

^1^Charité – Universitätsmedizin Berlin, corporate member of Freie Universität Berlin and Humboldt-Universität zu Berlin, Department of Surgery, Campus Charité Mitte and Campus Virchow-Klinikum, 13353 Berlin, Germany

^2^ Berlin Institute of Health at Charité-Universitätsmedizin Berlin, Charitéplatz 1, 10117 Berlin, Germany

**Email of corresponding author:** [linda.feldbruegge@charite.de](mailto:linda.feldbruegge@charite.de)

**Supplementary Fig.1.** Covariate balance in both groups (use of intermittent Pringle maneuver vs. no use of intermittent Pringle maneuver), before (red) and after (blue) propensity score matching.


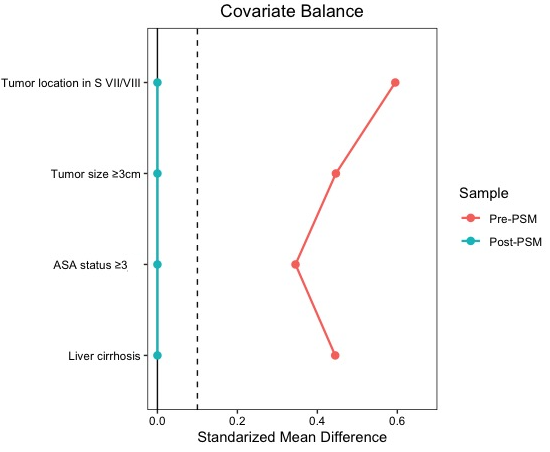


**Supplementary Fig. 2.** Correlation of serum levels of aspartate aminotransferase on postoperative day one (POD1) with duration of IPM.


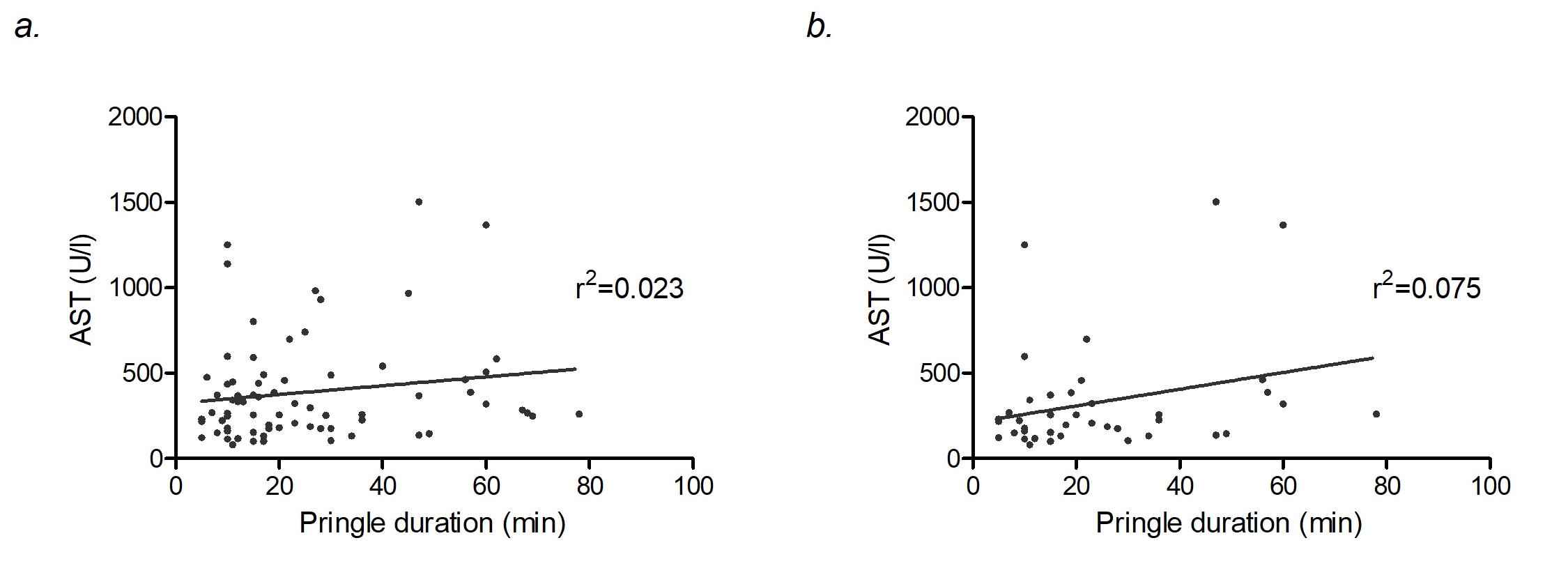
*a.* All patients; *b.* Subgroup of patients with liver cirrhosis.

**
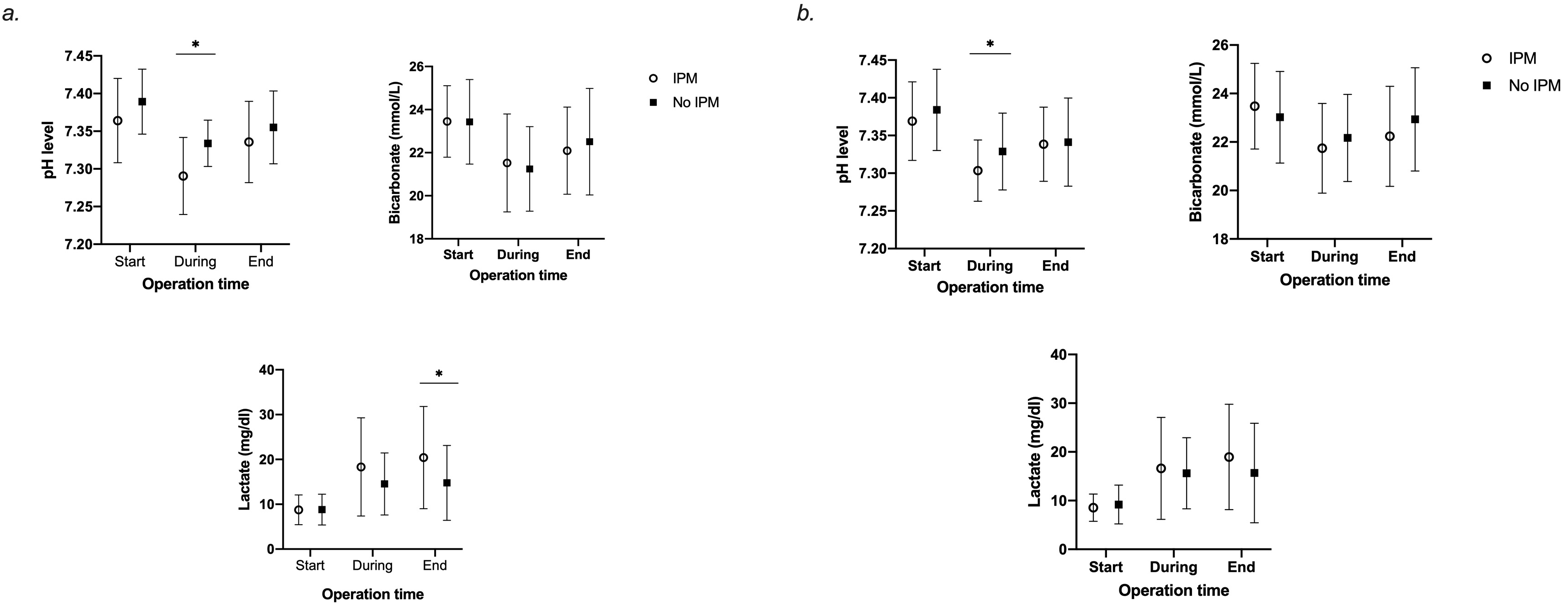
Supplementary Fig. 3.** Intraoperative arterial blood gas analysis at the beginning, during and at the end of minimally invasive liver surgery for hepatocellular carcinoma (HCC) with vs. without intermittent Pringle maneuver (IPM) *a.* All patients (after propensity score matching) *b.* Subgroup of patients with liver cirrhosis (after propensity score matching)

**Supplementary Table 1.** Intraoperative and postoperative outcome criteria after minimally invasive liver surgery for hepatocellular carcinoma (HCC) in the subgroup of patients with major resections with vs. without use of intermittent Pringle maneuver (IPM).

|  | **IPM**  **n = 19** | **No IPM**  **n = 14** | ***p*** |
| --- | --- | --- | --- |
| Duration of surgery (min) | 363 (195-491) | 314 (143-461) | 0.091 |
| Red blood cell transfusion | 1 (5%) | 1 (7%) | 1.000 |
| Textbook outcome ^a^ | 13 (69%) | 12 (86%) | 0.416 |
| Postoperative complications ^b^ | 12 (63%) | 9 (64%) | 0.947 |
| Severe complications ^b, c^ | 6 (32%) | 2 (14%) | 0.416 |
| PHLF | 0 | 1 (7%) | 0.424 |
| Grade A | 0 | 1 (100%) |  |
| Grade B | 0 | 0 |  |
| Bile leak | 4 (21%) | 4 (29%) | 0.695 |
| Post hepatectomy hemorrhage | 0 | 1 (8%) | 0.424 |
| Mortality ^b^ | 0 | 0 |  |
| LOS – ICU (days) | 2 (0-28) | 1 (1-5) | 0.114 |
| LOS – hospital (days) | 8 (6-61) | 8 (5-19) | 0.397 |
| R0 | 18 (95%) | 14 (100%) | 1.000 |
| Conversion rate | 0 | 0 |  |

Data presented as number (percent) for categorical or median (minimum-maximum) for continuous variables. PHLF: Post-hepatectomy liver failure [1]; LOS length of stay; ICU intensive care unit; R0 resection status (no residual tumor).

^a^ Defined as: No severe complication (≥3 according to Clavien-Dindo classification of postoperative complications) [2]; no intraoperative complication ≥2 (according to the Oslo classification of intraoperative complications [3]); R0 resection status achieved; no re admission in 30 days post discharge; no in-hospital mortality; absence of bile leak grade B or C [4].

^b^ within 90 days after surgery

^c^ ≥3a according to Clavien-Dindo classification of postoperative complications [2].

**Supplementary Table 2.** Intraoperative and postoperative outcome criteria after minimally invasive liver surgery for hepatocellular carcinoma (HCC) in the subgroup of patients with long total duration of intermittent Pringle maneuver (IPM ≥30 minutes) vs. without use of IPM.

|  | **long IPM**  **n = 22** | **No IPM**  **n = 78** | ***p*** |
| --- | --- | --- | --- |
| Duration of surgery (min) | 263 (113-455) | 191 (49-461) | *<0.001* |
| Red blood cell transfusion | 3 (14%) | 3 (4%) | 0.118 |
| Textbook outcome ^a^ | 17 (77%) | 65 (83%) | 0.536 |
| Postoperative complications ^b^ | 8 (36%) | 33 (42%) | 0.635 |
| Severe complications ^b, c^ | 3 (14%) | 10 (13%) | 0.920 |
| PHLF | 1 (5%) | 3 (4%) | 1.000 |
| Grade A | 0 | 2 (75%) |  |
| Grade B | 1 (100%) | 1 (25%) |  |
| Bile leak | 0 | 6 (8%) | 0.334 |
| Post hepatectomy hemorrhage | 1 (5%) | 2 (3%) | 0.530 |
| Mortality ^b^ | 1 (5%) | 0 | 0.220 |
| LOS – ICU (days) | 1 (0-43) | 1 (0-6) | 0.109 |
| LOS – hospital (days) | 6 (3-42) | 6 (3-26) | 0.833 |
| R0 | 21 (96%) | 75 (96%) | 1.000 |
| Conversion rate | 0 | 1 (2%) | 1.000 |

Data presented as number (percent) for categorical or median (minimum-maximum) for continuous variables. PHLF: Post-hepatectomy liver failure [1]; LOS length of stay; ICU intensive care unit; R0 resection status (no residual tumor).

^a^ Defined as: No severe complication (≥3 according to Clavien-Dindo classification of postoperative complications) [2]; no intraoperative complication ≥2 (according to the Oslo classification of intraoperative complications [3]); R0 resection status achieved; no re admission in 30 days post discharge; no in-hospital mortality; absence of bile leak grade B or C [4].

^b^ within 90 days after surgery

^c^ ≥3a according to Clavien-Dindo classification of postoperative complications [2].

**REFERENCES**

1. Rahbari NN, Garden OJ, Padbury R, Brooke-Smith M, Crawford M, Adam R, Koch M, Makuuchi M, Dematteo RP, Christophi C, Banting S, Usatoff V, Nagino M, Maddern G, Hugh TJ, Vauthey JN, Greig P, Rees M, Yokoyama Y, Fan ST, Nimura Y, Figueras J, Capussotti L, Büchler MW, Weitz J (2011) Posthepatectomy liver failure: a definition and grading by the International Study Group of Liver Surgery (ISGLS). Surgery 149**:**713-724

2. Dindo D, Demartines N, P-A. C (2004) Classification of surgical complications: A new proposal with evaluation in a cohort of 6336 patients and results of a survey. . Annals of surgery 204

3. Kazaryan AM, Røsok BI, Edwin B (2013) Morbidity assessment in surgery: refinement proposal based on a concept of perioperative adverse events. ISRN Surg 2013**:**625093

4. Görgec B, Benedetti Cacciaguerra A, Lanari J, Russolillo N, Cipriani F, Aghayan D, Zimmitti G, Efanov M, Alseidi A, Mocchegiani F, Giuliante F, Ruzzenente A, Rotellar F, Fuks D, D'Hondt M, Vivarelli M, Edwin B, Aldrighetti LA, Ferrero A, Cillo U, Besselink MG, Abu Hilal M (2021) Assessment of Textbook Outcome in Laparoscopic and Open Liver Surgery. JAMA Surg 156**:**e212064
